# Transcription box-3 protects human umbilical vein endothelial cells in a high-glucose environment through sirtuin 1/AKT signaling

**DOI:** 10.3892/mmr.2020.11237

**Published:** 2020-06-15

**Authors:** Zhanjiang Yu, Wei Zhang, Xiankun Zhang, Donghui Xu, Na Wang

**Affiliations:** 1 Department of General Surgery, The Third Affiliated Hospital of Qiqihar Medical University, Qiqihar, Heilongjiang 161000, P.R. China; 2 Department of Endocrinology, The Third Affiliated Hospital of Qiqihar Medical University, Qiqihar, Heilongjiang 161000, P.R. China; 3 Department of Laboratory, The Third Affiliated Hospital of Qiqihar Medical University, Qiqihar, Heilongjiang 161000, P.R. China; 4 Department of Psychology, Qiqihar Medical University, Qiqihar, Heilongjiang 161000, P.R. China

**Keywords:** transcription box-3, sirtuin 1, angiogenesis, migration, invasion, diabetes

## Abstract

The increasing burden of diabetes in low and middle-income countries is attributable to both genetic and epigenetic factors. Environmental- and lifestyle-associated changes are also considered to be important contributors to this disease. The resultant co-morbidities arising from micro-and macrovascular changes in diabetes are difficult to manage and are an economic burden. However, very little is known about the molecular mechanisms that drive this phenotype. The present study aimed to investigate the role of sirtuin 1 (SIRT1)- and transcription box-3 (TBX-3)-mediated regulation of endothelial dysfunction, given the significance of SIRT1 in glucose metabolism and the role of TBX-3 in the maintenance of cellular proliferation, senescence and apoptosis. Following the recruitment of adult patients with and without diabetes, both SIRT1 and TBX-3 expression was confirmed to be present in the sera of the patients with diabetes and the patients without diabetes; however, both SIRT1 and TBX-3 expression levels were higher in the sera of the patients with diabetes. Human umbilical vein endothelial cells (HUVECs) were further used for in vitro studies. Using TBX-3 and SIRT1 knockdown models, the cellular responses to proliferation, migration, invasion and tube formation were investigated using an MTS, cell cycle analysis, wound healing, Transwell and tube formation assay, respectively. Western blotting was also used to determine the downstream signaling pathways involved. The genetic knockdown of TBX-3 in hyperglycemic conditions significantly decreased the cellular proliferation, migration, invasion and angiogenesis of HUVECs. It was subsequently identified that TBX-3 mediated its effects through the activation of AKT and vascular endothelial growth factor (VEGF) signaling. However, the genetic knockdown of SIRT1 in the presence of TBX-3 overexpression and glucose failed to activate the AKT and VEGF signaling pathways. In conclusion, the results of the present study suggested that SIRT1 may positively regulate TBX-3 in endothelial cells, therefore, SIRT1 and/or TBX-3 may serve as potential novel biomarkers for disease progression.

## Introduction

The public health system in China is increasingly burdened by a disproportionate increase in type 1 and type 2 diabetes (T2D) (1). While the mortality rate of the diseases and their presentation differ significantly, the overall morbidity and associated mortality rates associated with both the diseases are increasing sharply (2); for example, the morbidity rate in the elderly population with type 2 diabetes was recorded as 21.4 and 24.8% in 2001 and 2010, respectively, in China (2,3). A multitude of factors, such as obesity, age, diet, physical activity, and genetic and epigenetic modifications have been identified to be responsible for the increasing disease burden in the Chinese population (4).

Region-specific studies of diabetes have reported an incidence rate of 1.01 per 100,000 person years across all age groups in China (5), with an increased incidence rate in males compared with females (6). On the other hand, the prevalence of T2D has increased to 9% in the last three decades (7) and it has been demonstrated to be associated with high rates of morbidity and mortality (8).

Diabetes-induced microvascular diseases, including retinopathy, neuropathy and nephropathy, and macrovascular diseases, such as cardiovascular disease, stroke and peripheral disease, often arise due to the inadequate maintenance of glycemic control (9). It has been hypothesized that microvasculopathy may give rise to macrovasculopathy through hypoxia and changes in the vasa vasorum (10); the changes in the vasa vasorum have been often associated with endothelial dysfunction (11); however, the molecular mechanisms driving endothelial cell plasticity under hyperglycemic stress have received little attention.

Transcription box-3 (TBX-3) is a T-box transcription factor with a known role in cell development, patterning, exit from the cell cycle and apoptosis (12). Given the significance of this transcription factor and its modifications over the expression levels of its target genes in the normally quiescent endothelial cells, the present study aimed to investigate whether TBX-3 had a role in mediating endothelial dysfunction and its associated vasculopathy. Additionally, TBX-3 has been previously reported to have a role in promoting adipocyte self-renewal (13). Therefore, the present study also aimed to determine whether the expression levels of *TBX-3*, the gene coding for TBX-3, were upregulated in endothelial cells in response to hyperglycemia, in addition to their contribution to the neotransformation of cells.

To the best of our knowledge, the present study was the first demonstration of TBX-3-mediated changes in *in vitro* endothelial cells in a hyperglycemic environment. In addition, the study further determined the molecular pathways of TBX-3-mediated endothelial dysfunction.

## Materials and methods

### 

#### Clinical samples

The present study was conducted in accordance with the Declaration of Helsinki (14) and approved by the Medical Ethics Committee of Qiqihar Medical University. Written informed consent was obtained from all recruited subjects. A total of 15 patients with diabetes (age, 40–60 years; sex, 7 males and 8 females) who had a T2D diagnosis for ≥10 years according to the WHO criteria (15) and 15 non-diabetic age-matched participants (sex, 7 males and 8 females) were recruited to the Department of Endocrinology, The Third Affiliated Hospital of Qiqihar Medical University (Qiqihar, China) from January 2015 to January 2016. The exclusion criteria were as follows: Missing clinical data, the prescription of oral medication and a pre-existing diagnosis of essential hypertension, other autoimmune diseases, thyroid disease, renal disease, psychosis, acute infectious disease, acute stage of myocardial infarction and stroke, type 1 diabetes and other specific forms of diabetes, such as chronic pancreatitis and steroid-induced diabetes. Blood glucose levels of all patients were controlled using insulin through a subcutaneous injection. Fresh blood samples (5 ml/sample) were obtained after an overnight fast and centrifugation at 2,000 × g at 4°C for 15 min. The separated serum was stored in liquid nitrogen immediately following centrifugation for use in subsequent analysis.

#### Gene Expression Omnibus (GEO) datasets

The GSE49524 dataset (16) was downloaded from the GEO database (https://www.ncbi.nlm.nih.gov/geo). This dataset comprised the gene expression data of three mothers with gestational diabetes and three normal participants. The data analysis was performed using GEO2R software (17).

#### Human umbilical vein endothelial cell (HUVECs) culture and transfection

HUVECs were purchased from The Cell Bank of Type Culture Collection of the Chinese Academy of Sciences. HUVECs were cultured in DMEM (Gibco; Thermo Fisher Scientific, Inc.), supplemented with 10% FBS (Gibco; Thermo Fisher Scientific, Inc.), 100 U/ml penicillin and 100 µg/ml streptomycin (Gibco; Thermo Fisher Scientific, Inc.), and maintained at 37°C in a humidified 5% CO_2_ atmosphere. These cells were from the umbilical cord vein (18). HUVECs were treated with different concentrations of glucose (0, 10, 20, 30 or 40 mM; Sigma-Aldrich; Merck KGaA) for 1 h at 37°C and then cultured for 24 h in the medium without glucose.

Small interfering (si)RNA targeting TBX-3 (5′-GAGGAUGUACAUUCACCCG-3′), sirtuin 1 (SIRT1; 5′-GATGAAGTTGACCTCCTCA-3′) and control siRNA (5′-UUCUCCGAACGAGUCACG-3′), and TBX-3 overexpression plasmids, were synthesized by Shanghai GenePharma Co., Ltd. The pcDNA3.1 plasmid (Invitrogen; Thermo Fisher Scientific, Inc.) was used for the generation of the overexpression plasmids; an empty plasmid was used as the control for the overexpression experiments. HUVECs were transfected with 10 nM siRNA or 1 µg/100 µl overexpression plasmid using Lipofectamine^®^ 2000 reagent (Invitrogen; Thermo Fisher Scientific, Inc.), according to the manufacturer's protocol. Following transfection for 48 h at 37°C, the cells were used for subsequent experiments.

#### Reverse transcription-quantitative PCR (RT-qPCR)

Total RNA from cultured HUVECs and serum samples was extracted using TRIzol^®^ reagent (Invitrogen; Thermo Fisher Scientific, Inc.), according to the manufacturer's protocol. cDNA was then reverse transcribed from 2 µg RNA using a commercial PrimeScript™ RT Reagent kit (Takara Biotechnology Co., Ltd.), according to the manufacturer's protocol. The following RT temperature protocol was used: 37°C for 15 min and 85°C for 5 sec, followed by maintenance at 4°C for qPCR. qPCR was subsequently performed using a SYBR Green Real-Time PCR Master mix (Applied Biosystems; Thermo Fisher Scientific, Inc.), according to the manufacturer's protocol, on a 7500 Fast Real Time PCR system (Applied Biosystems; Thermo Fisher Scientific, Inc.). The following thermocycling conditions were used for the qPCR: Initial denaturation for 2 min at 94°C; followed by 30 cycles of 94°C for 30 sec, annellation at 55°C for 30 sec, extension at 74°C for 1 min; and a final extension at 74°C for 5 min. The following primer sequences were used for the qPCR: *TBX-3* forward, 5′-TTCCACATTGTAAGAGCCAATG-3′ and reverse, 5′-CTTTGAGGTTCGTTGTCCCTAC-3′; *SIRT1* forward, 5′-TGGCAAAGGAGCAGATTAGTAGG-3′ and reverse, 5′-CTGCCACAAGAACTAGAGGATAAGA-3′; and *GAPDH* forward, 5′-GGGCTGCTTTTAACTCTGGT-3′ and reverse, 5′-TGGCAGGTTTTTCTAGACGG-3′. Expression levels were quantified using the 2^−ΔΔCq^ method (19) and analyzed using 7500 FAST software (version 1.4; Applied Biosystems; Thermo Fisher Scientific, Inc.), with the expression levels of the mRNA normalized to the endogenous control, GAPDH.

#### Western blotting

Total protein was extracted from HUVECs using ice-cold RIPA lysis buffer [50 mM Tris pH 7.4, 150 mM NaCl, 1% Triton X-100, 1% sodium deoxycholate, 0.1% SDS with proteinase/phosphatase inhibitors (Thermo Fisher Scientific, Inc.)]. Total protein was quantified using a bicinchoninic acid assay (Beyotime Institute of Biotechnology) and 20 µg protein/lane was separated via 10% SDS-PAGE. The separated proteins were subsequently transferred onto a polyvinylidene difluoride membrane (EMD Millipore) and blocked in 5% non-fat dried milk (Thermo Fisher Scientific, Inc.) for 1 h at room temperature. The membranes were incubated with the following primary antibodies purchased from Santa Cruz Biotechnology, Inc. overnight at 4°C: Anti-TBX-3 (1:500; cat. no. sc-166623); anti-p21 (1:500; cat. no. sc-6246); anti-p27 (1:500; cat. no. sc-1641); anti-vascular endothelial growth factor (VEGF; 1:500; cat. no. sc-7269); anti-SIRT1 (1:500; cat. no. sc-74504); anti-phosphorylated (p)-AKT (1:200; cat. no. sc-293125); anti-AKT (1:500; cat. no. sc-135829); and anti-GAPDH (1:1,000; cat. no. sc-365062). Following the primary antibody incubation, the membrane was washed 3 times with TBS-0.1% Tween and incubated with horseradish peroxidase-conjugated anti-rabbit (1:10,000; cat. no. sc-2357) or anti-goat (1:10,000; cat. no. sc-2354) secondary antibodies (Santa Cruz Biotechnology, Inc.) for 1 h at room temperature. Protein bands were visualized using an enhanced chemiluminescence kit (Thermo Fisher Scientific, Inc.) and the protein expression levels were analyzed using ImageJ software (version 1.45; National Institutes of Health).

#### MTS assay

HUVEC proliferation was analyzed using an MTS assay kit (Promega Corporation), according to the manufacturer's protocol. Briefly, 2×10^3^ HUVECs/well were seeded into 96-well plates for 24 h at 37°C and the medium was subsequently replaced with fresh DMEM, prior to the cells being cultured for another 3 days at 37°C. Following culturing, 20 µl MTS reagent was added to each well and the plates were incubated for 1 h at 37°C. The absorbance was measured at 490 nm using a microplate reader (Bio-Rad Laboratories, Inc.). Each experiment was performed for 6 replicates, independently three times.

#### Flow cytometric analysis of the cell cycle

A total of 2×10^6^ HUVECs/ml were collected by centrifugation (1,000 × g; 5 min; 4°C) and washed twice with PBS buffer, prior to being fixed with ice-cold 70% ethanol for 5 min at 4°C. The cells were incubated with 1 µg/ml RNase I (Sigma-Aldrich; Merck KGaA) at 37°C for 1 h in the dark. Following the addition of 20 µg/ml propidium iodide at 37°C for 15 min, the samples were analyzed using an LSR II flow cytometer (BD Biosciences). Cells were analyzed using FlowJo version 10.0 software (FlowJo LLC).

#### Wound healing assay

HUVECs (1×10^6^ cells/well) were seeded into six-well plates and cultured 37°C in a 5% CO_2_ incubator until they reached 100% confluence in DMEM, supplemented with FBS. Subsequently, an artificial homogenous wound was made in the cell monolayer using a sterile 200-µl plastic micropipette tip. The cells were cultured for a further 24 h in DMEM without FBS at 37°C. The wound closure area was visualized at 0 and 24 h post-wound creation using a light microscope (magnification, ×50; Nikon Corporation) and ImageJ software was used to measure the distance of cell migration.

#### Transwell invasion assay

HUVECs (2×10^5^ cells/well) were plated into the upper chambers of Transwell plates (8-µm pore size) in serum-free DMEM. Transwell membranes were precoated with Matrigel (Corning Inc.) for 30 min at 37°C. DMEM supplemented with 10% FBS was plated into the lower chambers. Following incubation for 24 h at 37°C in a 5% CO_2_ incubator, the invasive cells in the lower chamber were fixed with 100% methanol for 5 min at room temperature and stained with 0.1% crystal violet solution for 1 min at room temperature. Stained cells were counted in six randomly selected visual fields under a light microscope (magnification, ×100; Nikon Corporation) and ImageJ software was used to analyze the cell invasion.

#### Matrix metalloproteinases (MMPs) activity detection

A Fluorokine MAP human MMP2 and MMP9 kit (R&D Systems, Inc.) was used to detect MMP activity, according to the manufacturer's protocol. Briefly, HUVECs (2×10^5^ cells/well) were cultured in 24-well plates for 24 h at 37°C, and the suspension was collected and centrifuged for 15 min at 10,000 × g at room temperature. Active forms of MMP2 and MMP9 levels were measured at a wavelength of 340 nm/465 nm, respectively.

#### Tube formation

To analyze tube formation, 96-well plates were precoated with Matrigel (250 µg/ml; Corning Inc.) at 37°C for 30 min to solidify. Subsequently, 1×10^4^ HUVECs/well were seeded into the plates and incubated at 37°C for 4 h. The network-like structures ‘tubes’ were observed under a light microscope (magnification, ×25; Nikon Corporation) and ImageJ software was used to measure tube formation.

#### Statistical analysis

Statistical analysis was performed using SPSS version 19.0 software (IBM Corp.) and data are expressed as the mean ± SD. Statistical differences between groups were analyzed using an unpaired Student's t-test or a one-way ANOVA followed by a Bonferroni post hoc test for multiple comparisons. The correlation between *TBX-3* and *SIRT1* expression levels was determined using Pearson's correlation analysis. P<0.05 was considered to indicate a statistically significant difference. All experiments were repeated ≥3 times.

## Results

### 

#### Upregulation of TBX-3 in HUVECs and serum of patients with diabetes

**Figure 1.**
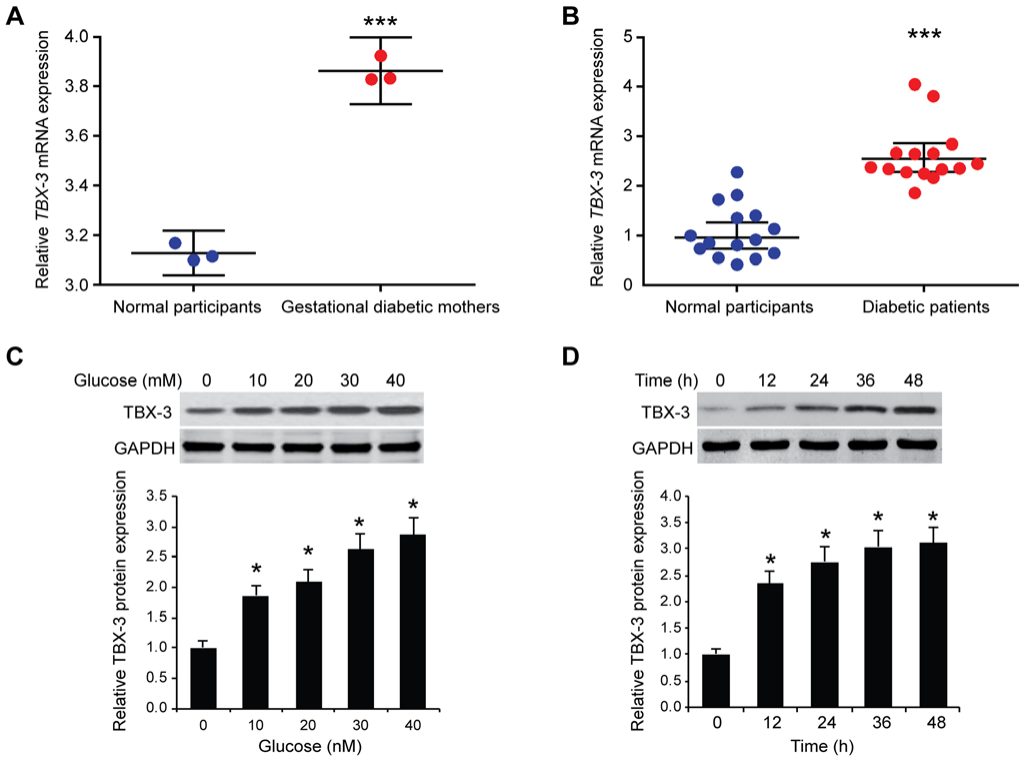
Upregulation of TBX-3 expression levels in HUVECs and the serum of patients with diabetes. (A) TBX-3 mRNA expression levels in primary endothelial cells from the umbilical cord vein between gestational diabetic and normal women obtained from the Gene Expression Omnibus dataset GSE49524. ***P<0.001 vs. normal participants. (B) TBX-3 mRNA expression levels in the serum of 15 patients with type 2 diabetes compared with 15 healthy participants. ***P<0.001 vs. normal participants. (C) Expression levels of TBX-3 were analyzed in HUVECs treated with different concentrations of glucose (0, 10, 20, 30 or 40 mM) using western blotting. *P<0.05 vs. 0 mM. TBX-3 expression levels were semi-quantified in the below panel. (D) Expression levels of TBX-3 were analyzed in HUVECs treated with 30 mM glucose at different time points (0, 12, 24, 36 or 48 h) using western blotting. TBX-3 expression levels were semi-quantified in the below panel. *P<0.05 vs. 0 mM. TBX-3, transcription box-3; HUVECs, human umbilical vein endothelial cells.

The differences in the mRNA expression levels of TBX-3 in primary endothelial cells from the umbilical cord vein between women with gestational diabetes and normal women was investigated. *TBX-3* mRNA expression levels were significantly upregulated in gestational diabetic women obtained from the Gene Expression Omnibus (GEO) dataset GSE49524 compared with normal participants (P<0.001; Fig. 1A). Similarly, the expression levels of *TBX-3* were identified to be significantly upregulated in the serum of 15 patients with diabetes compared with normal participants (Fig. 1B). Subsequently, HUVECs were treated with increasing concentrations of glucose (0, 10, 20, 30 or 40 mM). Western blotting results revealed that TBX-3 protein expression levels were significantly increased by glucose in HUVECs in a dose-dependent manner compared with the control group (P<0.05; Fig. 1C). Furthermore, HUVECs were treated with 30 mM glucose for different time points (0, 12, 24, 36 or 48 h); the western blotting results revealed that TBX-3 expression levels were increased by glucose in a time-dependent manner (P<0.05; Fig. 1D).

#### Effect of TBX-3 on cell proliferation under high-glucose conditions

**Figure 2.**
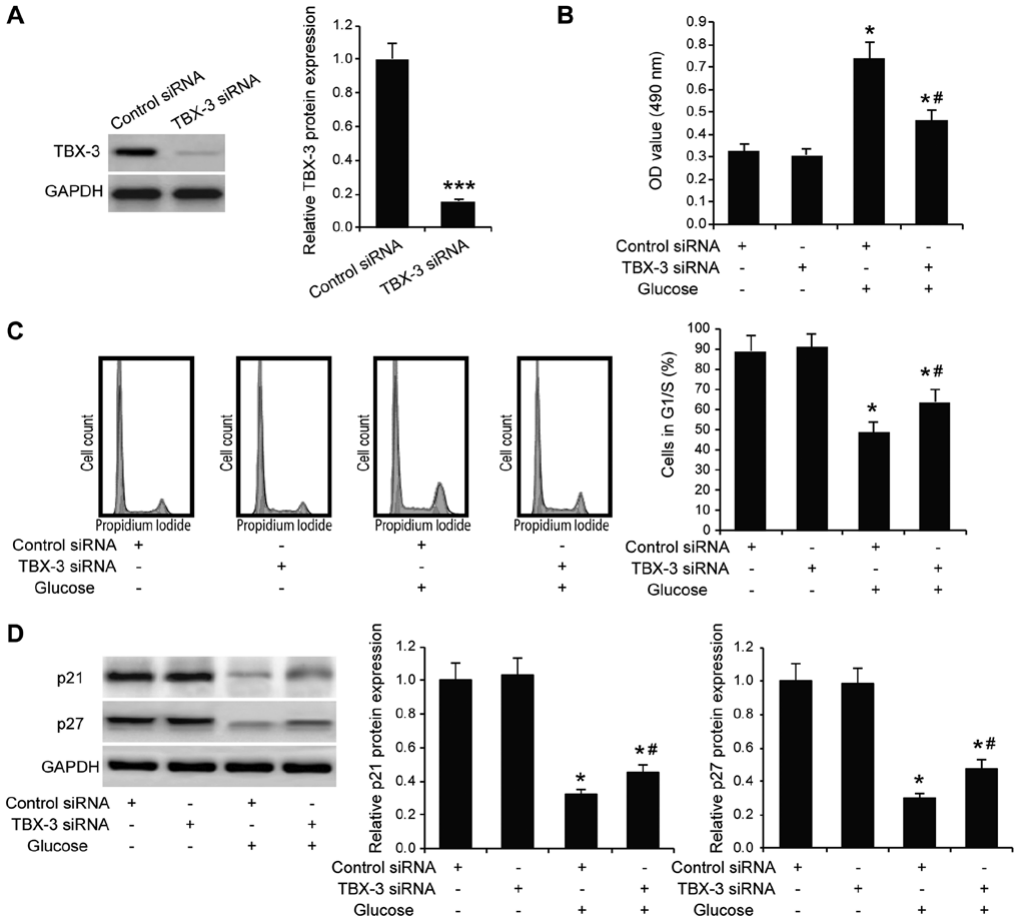
Effect of TBX-3 on cell proliferation induced by high glucose levels. (A) Western blotting was used to analyze the expression levels of TBX-3 in HUVECs transfected with TBX-3 siRNA or control siRNA. TBX-3 protein expression levels were semi-quantified in the right panel. ***P<0.001 vs. control siRNA. (B) MTS cell proliferation assay was performed in TBX-3 knockdown HUVECs treated with and without 30 mM glucose. *P<0.05 vs. control siRNA without glucose; ^#^P<0.05 vs. control siRNA with glucose. (C) Cell cycle analysis was performed in TBX-3 knockdown HUVECs treated with or without 30 mM glucose. The percentage of HUVECs in the G1/S phase was calculated. *P<0.05 vs. control siRNA without glucose; ^#^P<0.05 vs. control siRNA with glucose. (D) Western blotting was used to analyze the expression levels of p21 and p27 in HUVECs transfected with TBX-3 siRNA or control siRNA. TBX-3 protein expression was semi-quantified in the right panel. *P<0.05 vs. control siRNA without glucose; ^#^P<0.05 vs. control siRNA with glucose. TBX-3, transcription box-3; HUVECs, human umbilical vein endothelial cells; siRNA, small interfering RNA; OD, optical density.

It has been reported that glucose alone exerts effects on cell proliferation (20,21). To investigate whether TBX-3 affected HUVEC function in a high-glucose environment, TBX-3 expression was knocked down in HUVECs using siRNA transfection. The transfection efficiency of the siRNA was confirmed using western blotting (P<0.001; Fig. 2A). The TBX-3 knockdown HUVECs were subjected to an MTS cell proliferation assay; it was identified that TBX-3 knockdown did not affect HUVEC proliferation in a non-glucose environment (P≥0.05; Fig. 2B); however, upon treatment with 30 mM glucose, the genetic knockdown of TBX-3 significantly decreased the proliferation of HUVECs compared with the HUVECs transfected with control siRNA and treated with glucose (P<0.05; Fig. 2B). Cell cycle analysis further confirmed that TBX-3 knockdown significantly inhibited cell proliferation in a high-glucose environment (P<0.05; Fig. 2C). In addition, the results of western blotting analysis revealed that p21 and p27 expression levels were decreased in a high-glucose environment; however, the genetic knockdown of TBX-3 significantly increased the p21 and p27 expression levels compared with the HUVECs transfected with the control siRNA and treated with glucose (P<0.05; Fig. 2D).

#### Effect of TBX-3 on migration, invasion and angiogenesis induced by high glucose

**Figure 3.**
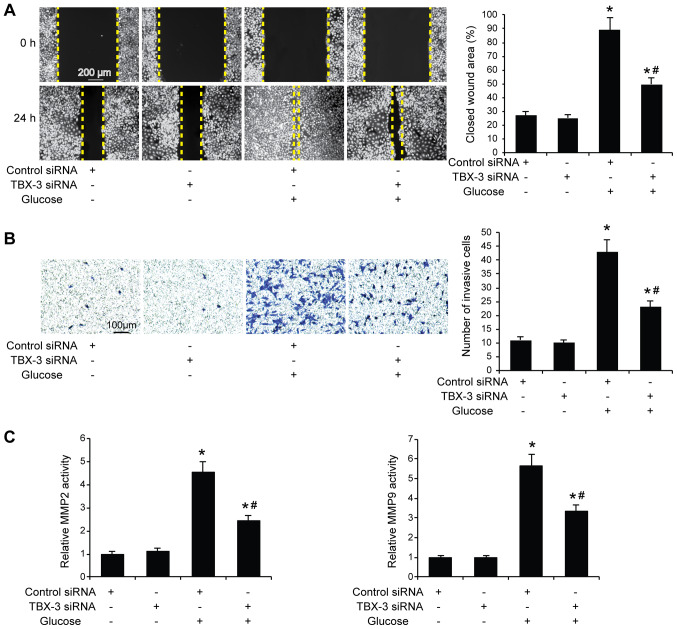
Effect of TBX-3 on cell migration and invasion induced by high glucose levels. (A) A wound healing assay was performed to analyze the migratory ability of TBX-3 knockdown HUVECs treated with and without 30 mM glucose. (B) Transwell assay was performed to analyze the invasive ability of TBX-3 knockdown HUVECs treated with and without 30 mM glucose. (C) MMP activity in TBX-3 knockdown HUVECs treated with or without 30 mM glucose. *P<0.05 vs. control siRNA without glucose; ^#^P<0.05 vs. control siRNA with glucose. TBX-3, transcription box-3; HUVECs, human umbilical vein endothelial cells; siRNA, small interfering RNA; MMP, matrix metalloproteinase.

**Figure 4.**
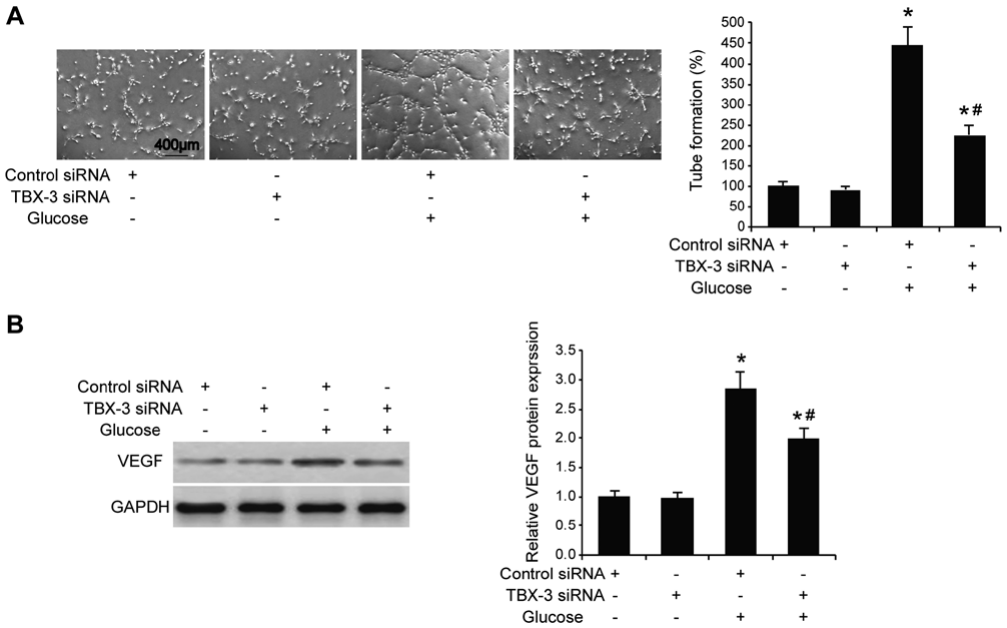
Effect of TBX-3 on angiogenesis induced by high glucose levels. (A) Tube formation assay was performed using TBX-3 knockdown HUVECs treated with and without 30 mM glucose. (B) Western blotting was used to analyze the expression levels of VEGF in TBX-3 knockdown HUVECs treated with and without 30 mM glucose. VEGF protein expression was semi-quantified in the right panel. *P<0.05 vs. control siRNA without glucose; ^#^P<0.05 vs. control siRNA with glucose. TBX-3, transcription box-3; HUVECs, human umbilical vein endothelial cells; siRNA, small interfering RNA; VEGF, vascular endothelial growth factor.

Subsequently, whether TBX-3 knockdown affected HUVEC migration, invasion and angiogenesis in a high-glucose environment was investigated. The migratory and invasive abilities of HUVECs were analyzed using wound healing and Transwell assays, respectively, with and without high glucose. The results demonstrated that the knockdown of TBX-3 in HUVECs significantly decreased their migratory and invasive abilities in a high-glucose environment compared with HUVECs transfected with control siRNA with glucose treatment (P<0.05; Fig. 3A and B). As expected, the genetic knockdown of TBX-3 significantly decreased the activity of MMP2 and MMP9 compared with HUVECs transfected with control siRNA with glucose treatment (P<0.05; Fig. 3C). The angiogenic potential of HUVECs was also investigated using a tube formation assay; the knockdown of TBX-3 significantly inhibited the number of tubes formed in a high-glucose environment compared with HUVECs transfected with control siRNA with glucose treatment (P<0.05; Fig. 4A). Furthermore, the knockdown of TBX-3 also significantly decreased the expression levels of VEGF in a high-glucose environment compared with HUVECs transfected with control siRNA with glucose treatment (P<0.05; Fig. 4B).

#### Correlation of the expression levels between TBX-3 and SIRT1 in the serum of patients with diabetes

**Figure 5.**
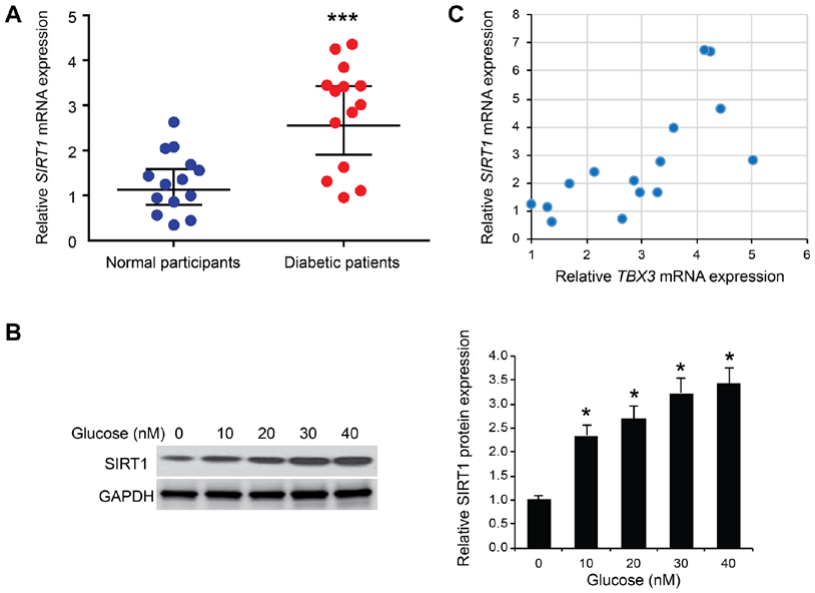
Positive correlation between the expression levels of TBX-3 and SIRT1. (A) SIRT1 mRNA expression levels in the serum of 15 patients with type 2 diabetes compared with 15 normal participants. ***P<0.001 vs. normal participants. (B) Expression levels of SIRT1 were analyzed using western blotting in HUVECs treated with different concentrations of glucose (0, 10, 20, 30 or 40 mM). SIRT1 protein expression levels were semi-quantified in the right panel. *P<0.05 vs. 0 mM. (C) Pearson's correlation analysis between SIRT1 and TBX-3 mRNA expression levels in the serum of 15 patients with type 2 diabetes. R=0.710; P<0.001. TBX-3, transcription box-3; HUVECs, human umbilical vein endothelial cells; siRNA, small interfering RNA; SIRT1, sirtuin 1.

The molecular regulation of TBX-3 function in glucose-affected HUVECs was further investigated. SIRT1 has been identified to serve an important role in glucose metabolism (22,23) and it was identified in one study to affect the expression levels of TBX-3 during the differentiation of stem cells (24). Thus, the expression levels of SIRT1 mRNA were determined in the serum of 15 patients with type 2 diabetes. The expression levels of SIRT1 mRNA were significantly increased in the serum of patients with diabetes compared with the normal participants (P<0.001; Fig. 5A). Subsequently, HUVECs were treated with different concentrations of glucose (0, 10, 20, 30 or 40 mM); the western blotting results revealed that SIRT1 protein expression levels were significantly increased by glucose in HUVECs in a dose-dependent manner (P<0.05; Fig. 5B). Subsequently, a positive correlation was observed between SIRT1 and TBX-3 mRNA expression levels in the serum of patients with diabetes (R=0.710; P<0.001; Fig. 5C), suggesting that SIRT1 may positively regulate TBX-3 in endothelial cells.

#### TBX-3 affects HUVECs through SIRT1-mediated AKT signaling

**Figure 6.**
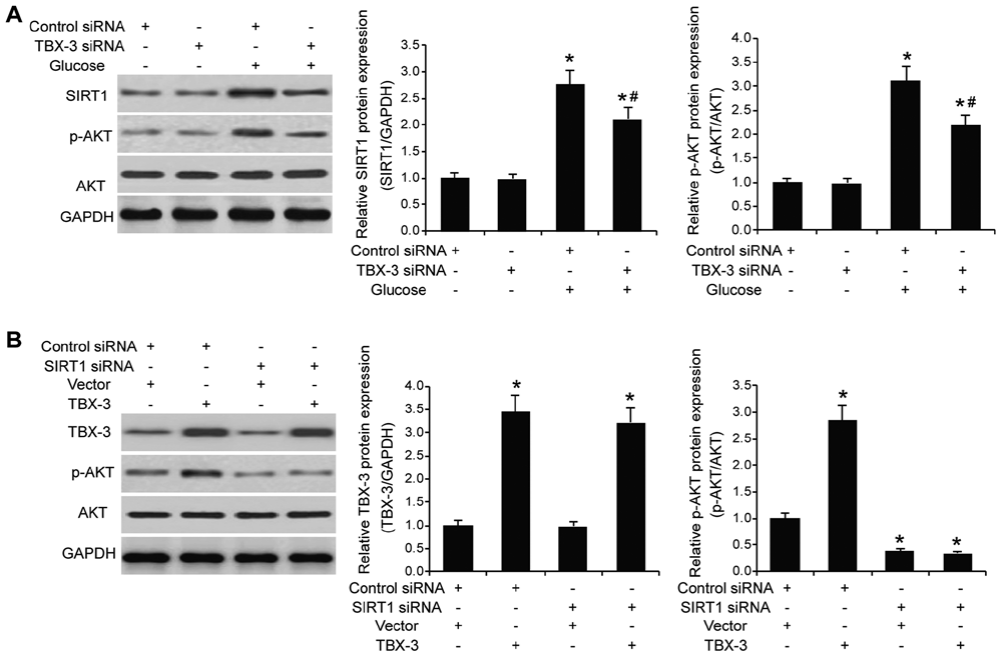
TBX-3 affects HUVECs through SIRT1-mediated AKT signaling. (A) Western blotting was used to analyze the expression levels of SIRT1, p-AKT and AKT in TBX-3 knockdown HUVECs treated with or without 30 mM glucose. The expression levels of SIRT1 and p-AKT protein were semi-quantified in the right panel. *P<0.05 vs. control siRNA without glucose; ^#^P<0.05 vs. control siRNA with glucose. (B) SIRT1 expression was knocked down in HUVECs by siRNA transfection and TBX-3 was then upregulated in HUVECs by overexpression plasmid transfection. Western blotting was used to analyze the expression levels of TBX-3, p-AKT and AKT in HUVECs treated with 30 mM glucose. Expression levels of TBX-3 and p-AKT protein were semi-quantified in the right panel. *P<0.05 vs. control siRNA and vector group. TBX-3, transcription box-3; HUVECs, human umbilical vein endothelial cells; siRNA, small interfering RNA; SIRT1, sirtuin 1; p-, phosphorylated

The expression levels of SIRT1 in TBX-3 knockdown HUVECs treated with and without high glucose were further investigated. The interference of TBX-3 expression affected SIRT1 expression levels; the genetic knockdown of TBX-3 significantly increased SIRT1 expression levels in a high glucose environment compared with the control siRNA group in a high glucose environment (P<0.05; Fig. 6A). As AKT signaling is downstream of SIRT1 (25), the expression levels of p-AKT and AKT in TBX-3 knockdown HUVECs treated with and without high glucose were subsequently investigated. The genetic knockdown of TBX-3 decreased the expression levels of p-AKT induced by high glucose (P<0.05; Fig. 6A); however, it had no effect on the total AKT expression levels (Fig. S1A). To investigate whether SIRT1 was required for TBX-3 function, the expression levels of *SIRT1* were knocked down using siRNA transfection (P<0.001; Fig. S2). The *SIRT1*-transfected cells were co-transfected to overexpress *TBX-3* concurrently (P<0.05; Fig. S3). The expression levels of p-AKT and AKT were subsequently determined using western blotting in a high-glucose environment (P<0.05; Fig. 6B and Fig. S1B). In HUVECs overexpressing *TBX-3*, whilst having genetically decreased levels of *SIRT1*, decreased expression levels of p-AKT were observed compared with the control group; however, total AKT expression levels in the HUVECs were not affected (Fig. S1B). These results indicated that SIRT1 may regulate TBX-3-driven AKT signaling.

## Discussion

Diabetes is a disease associated with high morbidity rates around the world, with an estimated prevalence of 9.3% (26), which commonly affects the younger population and inflicts associated co-morbidities, that are not only an economic burden but may also result in increased rates of mortality (27). Diabetes and its associated co-morbidities are often a burden on the overly stretched public health systems of low- and middle-income nations, such as China (27). The micro- and macrovascular changes in diabetes, which are associated with the endothelial dysfunction, have been identified to be regulators of its co-morbidities (10). Thus, the present study aimed to provide a molecular insight into the breach of the endothelial cell barrier under hyperglycemic conditions and the probable effects of this breach on endothelial cell survival and function.

The present study demonstrated that TBX-3 expression levels were increased in patients with diabetes compared with normal recruited controls. These increases in TBX-3 expression levels were associated with a concomitant increase in cellular proliferation. These results associated with the role of TBX-3 in promoting cellular proliferation are consistent with other studies; for example, in chondrosarcomas, it was identified that TBX-3 mediated cellular proliferation through the repression of p21 (28). Furthermore, a previous study in human embryonic stem cells demonstrated that the overexpression of TBX-3 was associated with increased cellular proliferation and an associated repression of NF-κB inhibitor β and p14 (29). Meanwhile, another study reported that the accelerated senescence of endothelial cells was mediated through AKT activation, p21 expression and p53 accumulation (30). These data are discordant with our present results, whereby the overexpression of TBX-3 resulted in the activation of AKT signaling. However, increased expression levels of p-AKT were not observed in SIRT1 knockdown cells following the introduction of the TBX-3 overexpression plasmid, which indicated that SIRT1 may be required for TBX-3 function.

It has also been previously demonstrated that TBX-3 expression levels were increased in the G1-phase, rising to their peak in the S-phase of the cell cycle; the presence of c-Myc in the S-phase was revealed to be responsible for TBX-3 upregulation both transcriptionally and translationally (31). In addition, c-Myc was identified to have a binding site in the promoter region of TBX-3 and its occupancy was revealed to be enhanced 600-fold during the S-phase; however, a minimal occupancy of the promoter region by c-Myc in the cells in the G1- and G2-phase was observed (31). These data supported our results that there were significantly fewer cells in the G1-phase in the presence of glucose alongside increased expression levels of TBX-3. The partial rescue of the cellular phenotype observed in the presence of TBX-3 siRNA and glucose may likely be due to the leaky expression of TBX-3.

TBX-3 has also been identified to regulate VEGF expression, which is known to be responsible for mediating endothelial cell migration as a pre-requisite to angiogenesis (32). VEGF mediates its action through promoting the activation and consequently, the differentiation of endothelial cells into stalk and phalanx cells, thus forming the body of the sprout and a tip cell phenotype. Tip cells are specialized filopodial extensions of endothelial cells stimulated by VEGF (33). Previous studies have also reported the involvement of transferrin, a protein, in endothelial cell migration, angiogenesis and neo-vascularization (34). In addition, epigenetic modifications through long non-coding RNA were found to be negatively correlated with the regulation of microRNA, which subsequently influenced the proliferation, migration and invasion of hemangioma-derived endothelial cells (35).

Furthermore, the present study revealed a positive correlation between TBX-3 and SIRT1 expression levels in endothelial cells. Contrary to the results of the present study, a previous study in human embryonic stem cells demonstrated that the increased expression levels of SIRT1 led to the downregulated expression levels of TBX-3 and other genes involved in development (24). Downregulated expression levels of SIRT1 and TBX-3 have also been observed in lung specimens derived from chronic obstructive pulmonary disease, which indicated that SIRT1 and TBX-3 are not always negatively regulated (36). The SIRT1/TBX-3 axis is microenvironment-dependent and undergoes function-driven regulation (37). The results of a previous in vivo study were consistent with the data from the present study, observing the overexpression of SIRT1 in response to hyperglycemia; the overexpression of SIRT1 significantly decreased the expression levels of senescence-associated markers, such as p53, p21 and plasminogen activator inhibitor-1 (38).

The failure to activate AKT in the absence of SIRT1 expression is also consistent with the hypothesis that SIRT1 binds to the promoter region of the TBX-3 gene and regulates transcription (37). AKT is under transcriptional control of TBX-3, therefore the TBX-3-mediated activation of AKT may be responsible for modulating the expression of VEGF through the activation of specificity protein 1; however, alternative mechanisms may also be plausible.

In conclusion, the present study hypothesized that the tolerance to hyperglycemia, induction of cellular proliferation, migration, invasion and angiogenesis in endothelial cells may not be just a pathognomonic feature of diabetes but may be a well-curated event with multiple molecular players. TBX-3 was identified to be an important factor; TBX-3-mediated AKT activation resulted in VEGF induction, which in turn was considered responsible for the angiogenic phenotype of endothelial cells. It was suggested that the TBX-3 expression levels in endothelial cells may be regulated by SIRT1; as the genetic knockdown of these genes resulted in partial or complete phenotype reversal, it could be suggested that endothelium-targeted TBX-3 therapy may serve as a therapeutic option for regulating diabetes-associated co-morbidities arising from endothelial dysfunction. As the delivery of gene therapy remains a challenge, TBX-3 may also be used as an early biomarker for disease prognosis and prevention.

## Supplementary Material

Supporting Data

## Data Availability

The datasets used and/or analyzed during the current study are available from the corresponding author on reasonable request.
