# Supplementary material for: Transcription box-3 protects human umbilical vein endothelial cells in a high-glucose environment through sirtuin 1/AKT signaling
Source: Mol Med Rep. 2020 Jun 15;22(2):1145–54. doi: 10.3892/mmr.2020.11237 (PMC7339771; doi:10.3892/mmr.2020.11237)

Figure S1. TBX-3 affects HUVECs through SIRT1-mediated AKT signaling. (A) Western blotting was used to analyze the expression levels of SIRT1, p-AKT and AKT in TBX-3 knockdown HUVECs treated with or without 30 mM glucose. Expression levels of AKT protein were semi-quantified. (B) SIRT1 expression was knocked down in HUVECs by siRNA transfection and TBX-3 was then upregulated in HUVECs by overexpression plasmid transfection. Western blotting was used to analyze the expression levels of TBX-3, p-AKT and AKT was performed in HUVECs treated with 30 mM glucose. Expression levels of AKT protein were semi-quantified. TBX-3, transcription box-3; HUVECs, human umbilical vein endothelial cells; siRNA, small interfering RNA; SIRT1, sirtuin 1; p-, phosphorylated.

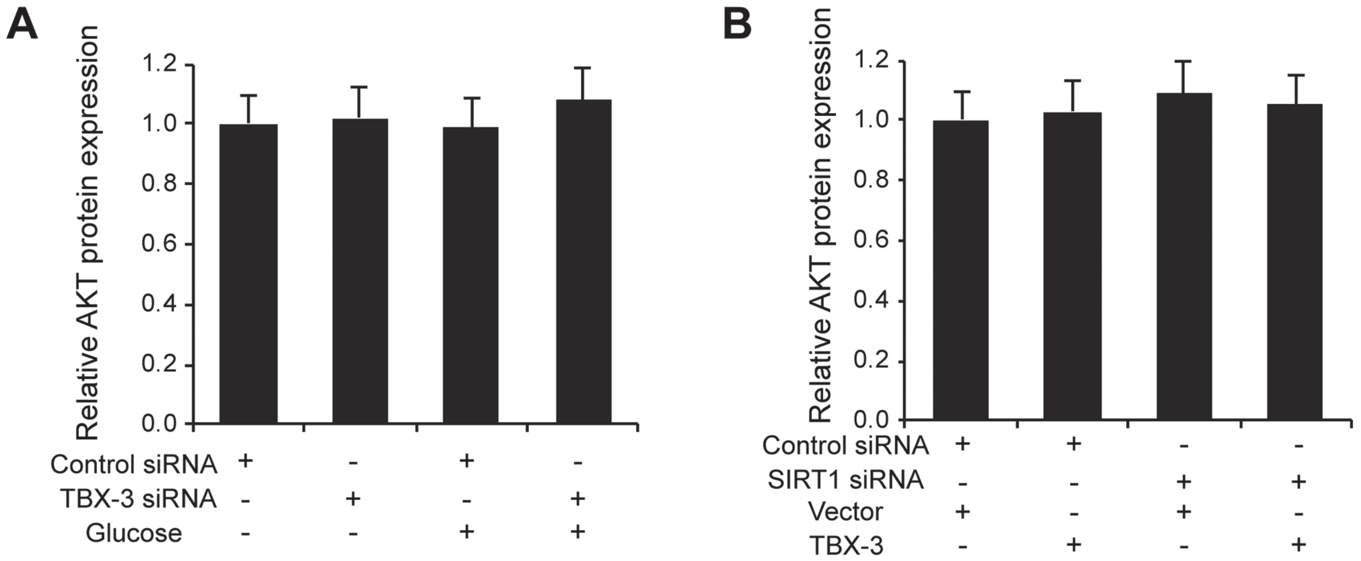

Figure S2. SIRT1 knockdown in HUVECs. Western blotting was used to determine the transfection efficiency of HUVECs transfected with SIRT1 siRNA or control siRNA. SIRT1 protein expression levels were semi-quantified in the right panel. ***P<0.001 vs. control siRNA. HUVECs, human umbilical vein endothelial cells; siRNA, small interfering RNA; SIRT1, sirtuin 1.

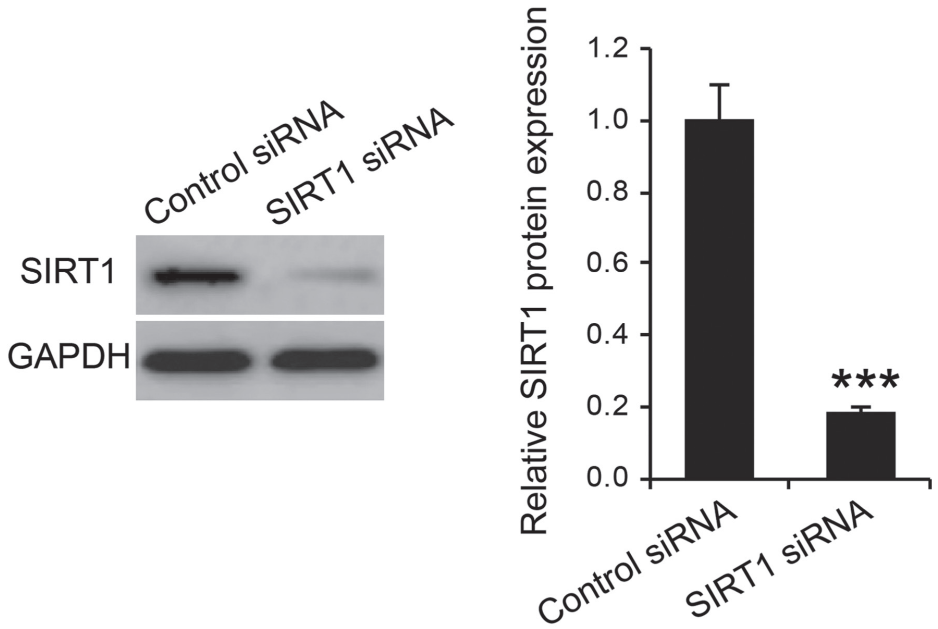

Figure S3. TBX-3 overexpression in HUVECs. Western blotting analysis was used to determine the transfection efficiency of HUVECs transfected with TBX-3 overexpression plasmid or empty vector. TBX-3 protein expression levels were semi-quantified in the right panel. *P<0.05 vs. vector. TBX-3, transcription box-3; HUVECs, human umbilical vein endothelial cells.

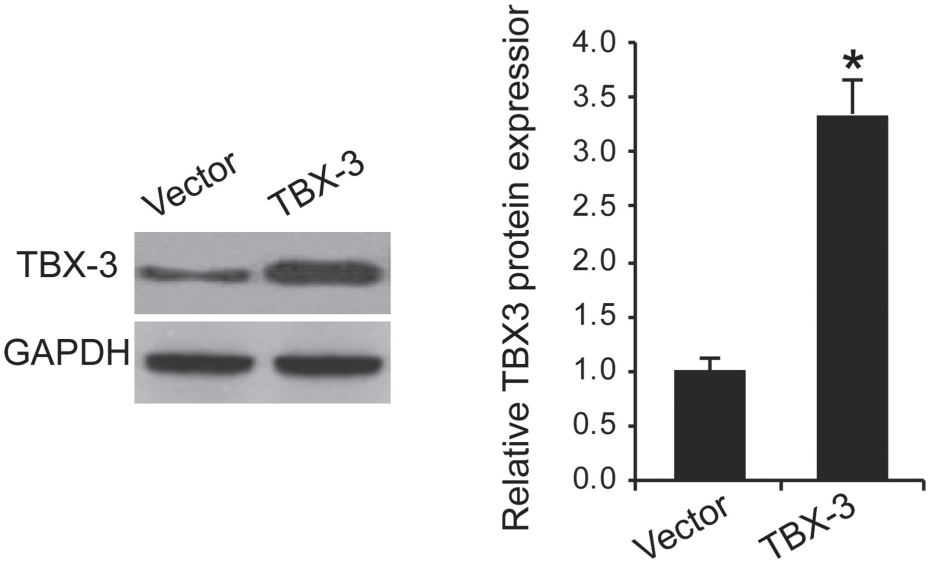

Supplement: Supporting Data [file Supplementary_Data.pdf]
